# Peculiar Presentation of Ulcerative Colitis

**DOI:** 10.1155/2016/1763041

**Published:** 2016-03-06

**Authors:** Hamdy M. A. Ahmed, Amany Diab, Ayman Ahmed, Samar Abohamad, Hala Elgendy

**Affiliations:** ^1^Clinical Immunology and Rheumatology Unit, Department of Medicine, Cairo University Hospitals, Cairo 11562, Egypt; ^2^Rochester General Hospital, Rochester, NY 14621, USA

## Abstract

Ulcerative colitis (UC) is a chronic inflammatory and recurrent disorder that is characterized by bowel inflammation. Among the extraintestinal manifestations (EIMs) that associate UC are the joints and renal manifestations. Joint affection in the form of arthritis can precede the intestinal manifestations of UC. However, renal affection with amyloidosis does not precede the UC diagnosis. Herein, we report a case of 26-year-old male diagnosed with UC after having peripheral arthritis for long time in addition to spondylitis and kidney amyloidosis.

## 1. Introduction

Ulcerative colitis (UC) is one of two types of inflammatory bowel disease (IBD). Its main presentation is episodic periods of a variety of symptoms such as abdominal pain, bleeding per rectum, and diarrhea along with periods of remission [1]. Diagnosis of UC is established by laboratory tests, endoscopic, histologic, and radiologic examinations. Medical treatment of UC differs according to the severity and extent of the disease and includes 5-aminosalicylates, corticosteroids, immunosuppressants, and anti-TNF alpha agents [2, 3]. Extraintestinal manifestations (EIMs) are seen in 25–40% of UC patients [4]. Arthritis, as a part of EIM, can affect the axial, peripheral joints, or a combination of both [5]. UC is an uncommon cause of secondary amyloidosis; however, it can have different renal presentations ranging from asymptomatic proteinuria to nephrotic syndrome [6].

## 2. Case Report

A 26-year-old man presented to the emergency department complaining of multiple swollen and painful joints. He had a five-year history of arthritis affecting the peripheral joints which started in the distal interphalangeal joints (DIPs), proximal interphalangeal joints (PIPs), metacarpophalangeal joints (MCPs), wrists, ankles, elbows, knees, hips, and shoulders. He did not seek medical advice until two years ago when laboratory results revealed no abnormalities with negative ANA, anti-DNA, and RF and joints X-rays were normal. He was started on nonsteroidal anti-inflammatory drugs that were not sufficient to control his symptoms and prednisolone was added. He was not compliant with his medications or follow-ups.

Three months before this admission, he reported worsening of pain in the same joints along with mild lower back pain for which he was started on corticosteroids, hydroxychloroquine, and methotrexate but his pain did not improve. He also reported self-limited mild diarrheal attacks without blood or mucous every 1 or 2 weeks associated with abdominal discomfort. He has no history of morning stiffness, oral ulcers, hair loss, photophobia, eye or mouth dryness, rashes, dyspnea, cough, expectoration, or urinary symptoms. His family history is not significant.

On examination, his vital signs were within normal range. Physical exam revealed swollen and tender DIPs, PIPs, MCPs, elbows, shoulders, knees, and ankles. Sacroiliac joints showed tenderness with limited movements of the lumbosacral vertebrae along with positive Modified Schober's Test. Also, mild bilateral lower limbs edema limited to his feet was noticed.

Laboratory investigations revealed normal complete blood picture and normal chemistry panel apart from albumin: 2 gm/dL (*N*: 3.5–5), total protein: 5.4 gm/dL (*N*: 6.6–8.3), LDH: 1310 U/L (*N*: 10–247), calcium: 8 mg/dL (*N*: 8.8–10.6), and phosphorus: 5.5 (*N*: 2.5–4.5). Also, his ESR (1st hour): 131 mm/hour, CRP: positive, and urine analysis revealed no abnormality except for albuminuria, A/C ratio of 5063 mg/gm. cr. ANA, RF, ENA, and HLAB27 were negative. C_3_ and C_4_ were normal.

**Figure 1:**
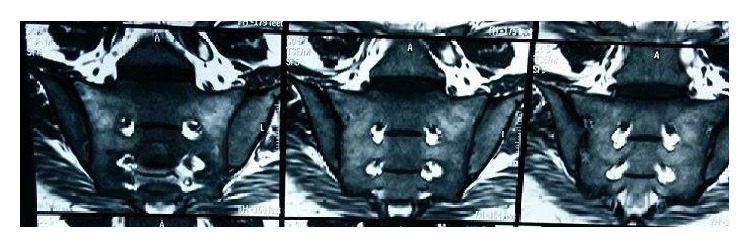
MRI of the sacroiliac joints with bilateral bone marrow edema.

**Figure 2:**
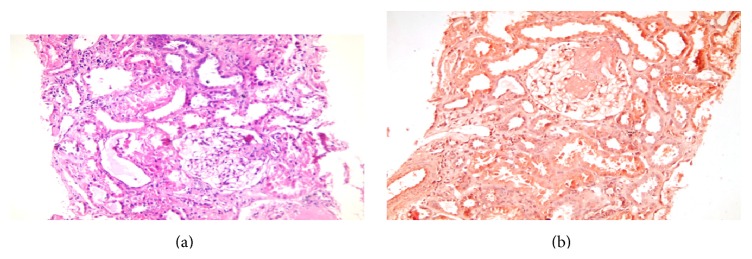
(a) Kidney biopsy (H&E stain). (b) Kidney biopsy (Congo red stain).

X-rays of both hands were normal while knees X-rays showed mild osteoarthritic changes. MRI of the sacroiliac joints showed bilateral marrow edema denoting sacroiliitis (Figure 1). Abdominal ultrasound revealed bilateral enlarged grade III pathological kidneys and bright hepatomegaly. Renal biopsy showed amyloidosis with mild fibrosis and mild interstitial nephritis (Figures 2(a) and 2(b)).

One month after admission, the patient developed an attack of bloody diarrhea. Colonoscopy showed extensive hyperemia and diffuse ulceration of the rectum, sigmoid, descending, and transverse coli along with hyperemia of terminal ileum. Histopathological examination of colonic biopsies showed chronic active colitis with cryptitis and chronic nonspecific ileitis. He was diagnosed with ulcerative colitis complicated with peripheral and axial arthritis and kidney amyloidosis and was started on steroids and sulfasalazine. Follow-ups later showed marked improvement of patient symptoms.

## 3. Discussion

The patient in this case was diagnosed to have UC by clinical symptoms, endoscopic and histological findings. Review of literature reveals that EIMs can be seen in 25–40% of IBD patients [4]. Inflammatory manifestations of the skin, eyes, liver, and joints are considered primary EIMs [7]. Arthritis related to IBD is considered a subset of the seronegative spondyloarthropathies. It can be axial, peripheral, or combination and may be symptomatic prior to IBD diagnosis in 10–30% of cases [4, 5]. Three types of peripheral arthritis have been described. Type I peripheral arthritis is pauciarticular and strongly associated with IBD activity and other EIMs. Type II peripheral arthritis is polyarticular and not usually associated with disease activity or other EIMs, with the exception of uveitis. Type III is rare and includes involvement of both peripheral and axial joints. Axial involvement includes spondylitis and sacroiliitis. Spondylitis can occur in 2–6% of patients with UC. Sacroiliitis can be asymptomatic and are increasingly recognized due to improvements in the sensitivity of magnetic resonance imaging (MRI) [5, 8]. Accordingly, this patient had type III peripheral arthritis, as he had both peripheral arthritis five years prior to UC diagnosis and axial involvement occurring a few months before UC diagnosis. Since he had lower limb edema, hypoalbuminemia, heavy proteinuria, and large kidneys, a renal biopsy was done and revealed renal amyloidosis. Inflammatory bowel disease is an uncommon cause of secondary amyloidosis with a prevalence of 0–0.4% in UC with a lower prevalence clinically and a higher prevalence at autopsy [6, 9]. The time from diagnosis of IBD to the diagnosis of amyloidosis ranged from 0 to 28 years [10]. Kidney stones, enterovesical fistulas, and ureteral obstruction are the most common EIMs that affect 4–23% of IBD patients. Glomerulonephritis (GN) ranges from minimal change to rapidly progressive crescentic GN. Tubulointerstitial abnormalities are more detected in autopsy studies [6, 11]. Due to limited affordability of anti-TNF alpha drugs and accepted kidney functions, sulfasalazine was used in this case. Sulfasalazine should not be used in patients with severe renal impairment and should be used very cautiously in patients with moderate renal impairment with oliguria [12].
